# Predicting low-concentration effects of pesticides

**DOI:** 10.1038/s41598-019-51645-4

**Published:** 2019-10-24

**Authors:** Matthias Liess, Sebastian Henz, Saskia Knillmann

**Affiliations:** 1 UFZ - Helmholtz Centre for Environmental Research, Dept. of System-Ecotoxicology, Permoserstrasse 15, D-04318 Leipzig, Germany; 2 RWTH Aachen University, Institute for Environmental Research (Biology V), Worringerweg 1, D-52074 Aachen, Germany

**Keywords:** Environmental impact, Freshwater ecology

## Abstract

We present a model to identify the effects of low toxicant concentrations. Due to inadequate models, such effects have so far often been misinterpreted as random variability. Instead, a tri-phasic relationship describes the effects of a toxicant when a broad range of concentrations is assessed: i) at high concentrations where substantial mortality occurs (LC_50_), we confirmed the traditional sigmoidal response curve (ii) at low concentrations about 10 times below the LC_50_, we identified higher survival than previously modelled, and (iii) at ultra-low concentrations starting at around 100 times below the LC_50_, higher mortality than previously modelled. This suggests that individuals benefit from low toxicant stress. Accordingly, we postulate that in the absence of external toxicant stress individuals are affected by an internal “System Stress” (SyS) and that SyS is reduced with increasing strength of toxicant stress. We show that the observed tri-phasic concentration-effect relationship can be modelled on the basis of this approach. Here we revealed that toxicant-related effects (LC_5_) occurred at remarkably low concentrations, 3 to 4 orders of magnitude below those concentrations inducing strong effects (LC_50_). Thus, the EC_x-SyS_ model presented allows us to attribute ultra-low toxicant concentrations to their effects on individuals. This information will contribute to performing a more realistic environmental and human risk assessment.

## Introduction

Concentration-response relationships are generally described with a log-logistic relationship where the response of organisms to toxicants increase monotonically with increasing exposure. For *Daphnia magna*, a common aquatic test species, this relationship results in a range of effect concentrations from high mortality (LC_50_) to practically undetectable mortality (LC_5_) with a factor generally below 10^1^. Accordingly, it is assumed that the effect threshold of toxicants is about one order of magnitude below the concentration at which strong effects occur. However, several investigations show at concentrations of approximately one order of magnitude below the LC_50,_ an increase in survival compared to the control^2^. The concept of the monotonic dose-response relationship was therefore expanded already more than 100 years ago. In 1888, the pharmacologist Hugo Schulz published a set of experiments showing that yeast cells considerably increased their activity when exposed to low concentrations of various toxicants^3^. This results in a bi-phasic concentration response, increasing survival and activity at low concentrations (hormesis), and decreasing survival and activity at high concentrations. Towards the end of the 20^th^ century, an increasing number of similar observations were published involving various toxicants and species. For example, in comparison to control conditions, low concentrations of petroleum hydrocarbons caused an increased survival in crab zoeae (*Rhithropanopeus harrisii*)^4^. In the following century, Edward Calabrese provided a multitude of examples of such hormetic concentration-response relationships^2^. Additionally, mathematical approaches were identified to fit the shape of these concentration-response relationships^5^. Interestingly, even stressors other than toxicants may also enhance the performance of organisms at low stress intensities. One example is a parasite-induced increase in salinity tolerance for the freshwater shrimp *Gammarus roeseli*^6^.

However, there is another anomaly in the concentration-effect relationship in studies investigating the effects of even lower toxicant concentrations. The few studies that also investigate ultra-low concentrations in relation to their effects, two or more orders of magnitude below the LC_50_ show that adverse toxicant effects may be present even below hormetic concentrations. For example, in a single-species microcosm, Ephemeroptera (*Cloeon dipterum*) experienced a significant 10% decrease in survival 4 orders of magnitude below the LC_50_^7^. Also, caddisflies (*Limnephilus lunatus*) showed a decrease in survival 4 orders of magnitude below the LC_50_^8,9^. A meta-study investigating the concentration-effect relationship of various toxicants with a concentration range of about 2 orders of magnitude found that out of 26 studies more than two thirds of studies (18) showed a sub-hormetic reduction in survival^1^. When such low concentrations of toxicants are tested, a tri-phasic concentration-response relationship becomes apparent with increasing toxicant concentrations: strong mortality at high concentrations, increasing survival at low, hormetic concentrations and small adverse effects at ultra-low subhormetic concentrations.

Our aim is to identify a first principle-based quantification of such tri-phasic cause-response relationships. We expect that such an approach is the key to identify and predict biological responses in the range of low and ultra-low concentrations.

## Results

### Experiments

**Figure 1:**
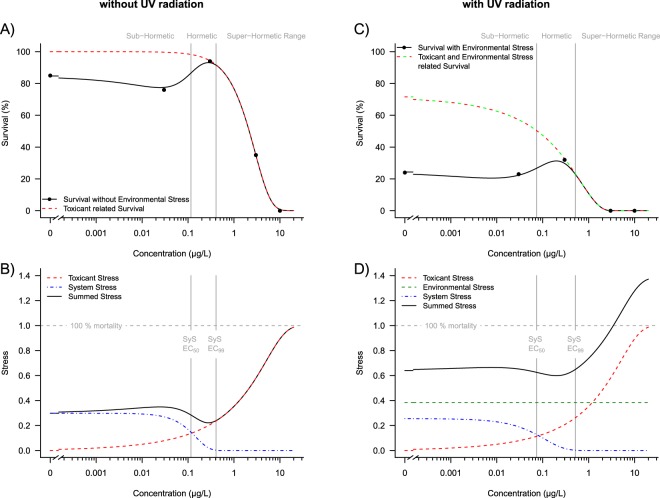
Tri-phasic concentration-response relationship. (**A)** Survival, no additional environmental stress. Dots relate to the average of measured effects (see methods) (**B)** Related stressors to experiment “without UV radiation”. (**C)** Survival with UV radiation as environmental stress. (**D)** Related stressors to experiment “with UV radiation”. Red lines represent toxicant stress, blue lines “System Stress” (SyS), green lines environmental stress.

We performed 6 concentration-response experiments pulse exposing the individually-kept crustacean *Daphnia magna* to the insecticide esfenvalerate for 24 h at day 8 after birth, then observed survival of individuals in clean water for an additional 21 days. Of these experiments, two involved exposure to esfenvalerate as the only stressor. For the other four experiments, the organisms were exposed to esfenvalerate in addition to three different environmental stressors for the whole duration of the experiment: high and low UV radiation, low food and high temperature (Supplementary Information, Fig. SI1). Figure 1 exemplarily shows the concentration-response relationship without environmental stress (Fig. 1A) and with UV radiation as an additional environmental stress (Fig. 1C). All 6 experiments revealed a tri-phasic concentration-response relationship: at low toxicant concentrations (0.03 µg/L) slight mortality compared to the control (1^st^ phase), at medium concentrations (0.3 µg/L) lower effects compared to the control (2^nd^ phase), and at high concentrations (>1 µg/L) the traditional sigmoidal response curve (3^rd^ phase). When additional constant environmental stressors are present, mortality increases in the control, but the general shape of the stress-response relationship remains.

### The Tri-phasic concentration-response relationship and system stress (SyS)

Based on the empirical observations, we postulate the existence of an internal “System Stress” (SyS), occurring at low to medium toxicant concentrations. This approach allows a mechanistic modelling of the stress response relationship with only three assumptions (Fig. 1A/C). The mathematical details are described in the Methods section.

1. Without, or at ultra-low pulsed toxicant stress, individuals develop internal SyS over time that may cause mortality of some individuals within a population. SyS develops regardless of the presence of constant environmental stress (Figs 1C, S1). Figure 2 shows that at 2 days after contamination, SyS is small compared to days 7 and 21. The SyS for each experiment is shown in the supporting information (Fig. S1).

**Figure 2:**
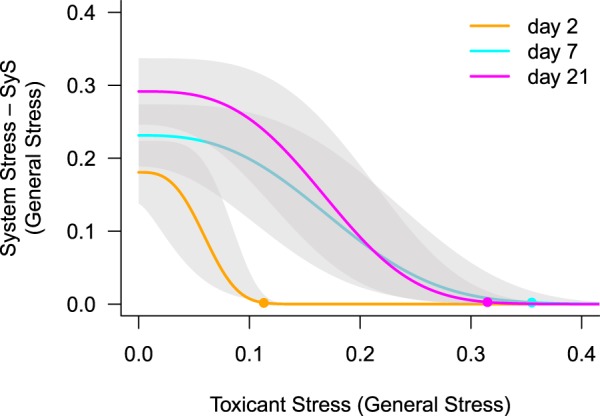
Relation of system stress (SyS) and toxicant stress. For all experiments, SyS increases from day 2 until day 21 when no toxicant stress is present (p = 0.017), with a significant difference between day 2 and day 21 (p = 0.015, ANOVA followed by Tukey’s HSD post hoc test) but not between day 2 and day 7. Increasing toxicant stress reduces SyS, fitted with a Weibull function based on the experiments displayed in the supplementary information. The toxicant stress where SyS approaches zero (EC_99_, dots) increases with time (p = 0.016) with significant differences between day 2 and day 7 (p = 0.011) and day 2 and day 21 (p = 0.030); ANOVA followed by Tukey’s HSD post hoc test.
2. Two opposing monotonic relationships are assumed: (i) toxicant stress increases with increasing toxicant concentration and (ii) SyS decreases with increasing toxicant stress close to 0 at low toxicant concentrations (here approximately 0.3 µg/L, Fig. 2). The relationship between SyS and pulsed toxicant stress can be described using a Weibull model (Fig. 1B/D).
3. All independent stressors - including SyS, toxicant stress and environmental stress, if present - are additive according to the “Stress Addition Model” (SAM)^1^, with the sum of the general stress determining the total stress exerted on an individual within a population. Notably, in comparison to the null-model of “concentration addition”^10^ or “effect addition”^11^, the SAM predicts the synergistic effects of independent stressors.

**Figure 3:**
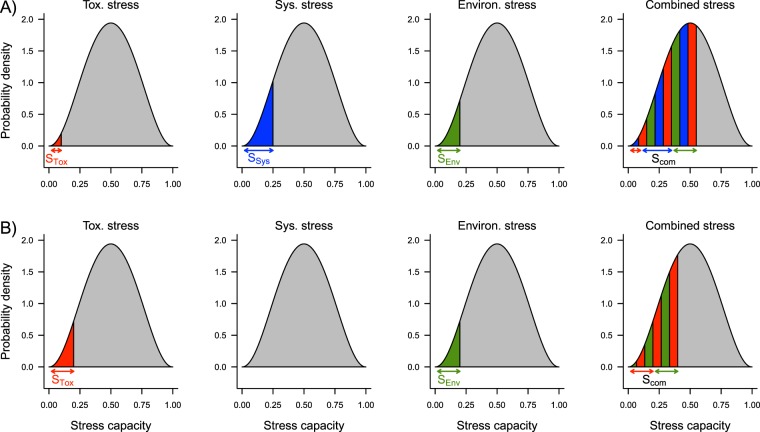
Two combined effect scenarios with and without system stress (SyS). (**A)** SyS present at ultra-low toxicant concentrations. (**B)** SyS not present between high and low, hormetic toxicant concentrations. For both scenarios, individual stress capacity within a population is beta distributed (grey area). Individuals with a stress capacity below the given stress level will die (coloured areas). Red – toxicant stress (S_Tox_), blue – system stress (S_SyS_), green – environmental stress (S_Env_), and striped – combined stress (S_Com_). The stress capacities of all three stressors are added, resulting in combined stress effects (see Methods for a mathematical description).
(iii) a further increase in toxicant stress reduces SyS to zero (Fig. 3B). Accordingly, at this hormetic concentration, there is no combined effect of SyS and toxicant stress. Therefore, at such low, hormetic toxicant concentrations, survival increases compared to that at sub-hormetic concentrations (all experiments resulted in a 9.3% increase in average survival compared to that at the sub-hormetic concentration, single sided t-test, p = 0.014);
(iv) high toxicant stress in the range of the LC_50_ reduces survival according to the well-known sigmoidal concentration-response relationship.

By combining these assumptions, the observed tri-phasic concentration-response relationship can be modelled:

(i) the observed mortality in the absence of toxicant and environmental stress is based on SyS alone (all experiments resulted in a 13.5% reduction in average survival, single sided t-test, p = 0.035);
(ii) at ultra-low toxicant stress, where SyS is still present, the SAM predicts a combined synergistic effect of SyS, toxicant stress and environmental stress (Fig. 3A). In comparison to the control this ultra-low toxicant stress at sub-hormetic concentrations causes a clear effect on survival (all experiments resulted in a 9.7% reduction in average survival compared to that of the control, single sided t-test, p = 0.020);

In some cases, the hormetic increase in survival is low compared to the sub-hormetic decrease in survival (Fig. S1C, food limitation) so that only a “step” in the concentration-effect relationship can be recognized. Nevertheless, this “step” can be described using the two opposing relationships regarding SyS and toxicant stress. A compensation of the costs of higher survival rates by toxicant effects in the hormetic concentration range on other endpoints could not be established. When comparing the relationship of SyS and toxicant stress for the experiments with and without environmental stress, the dependencies show a similar shape (Figs 1, S1). Also, offspring production in terms of number of neonates was highly correlated with individual mortality throughout all experiments, including those with environmental stress (r^2^ = 0.71, p < 0.001). Experiments with food deficiency did not follow the trend, as scarcely any neonates were produced due to low food conditions (Fig. S2).

### Predicting combined effects of environmental and toxicant stress

**Figure 4:**
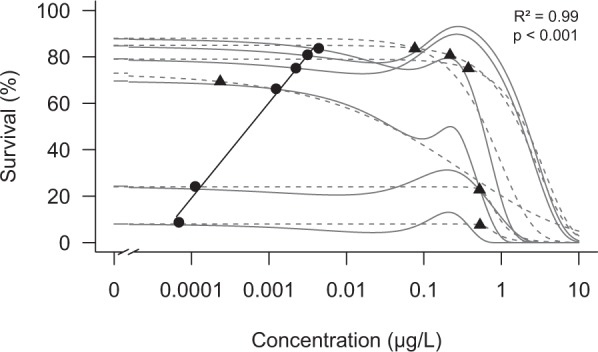
Tri-phasic concentration-response relationships modelled for all experiments. 21 days after short-term esfenvalerate exposure (solid lines) with EC_x-SyS_ modelled LC_5_ concentrations (dots). Linear regression between LC_5_ concentration and survival. Traditional monotonic concentration-response relationships modelled for all experiments (dashed line) with predicted LC_5_ concentrations (triangles). All experiments are shown in detail in Fig. S1.

A comparison of the traditional logistic-logistic concentration reaction model with the EC_x-SyS_ model showed that the new approach could be consistently better adapted to the observed effects (Table S2). The EC_x-SyS_ approach also revealed that increasing environmental stress results in greater toxicant-sensitivity of individuals related to small toxicant effects - the LC_5_ (R^2^ = 0.96, p < 0.001). In detail, weak environmental stress resulted in an esfenvalerate LC_5_ of approximately 0.003 µg/L, while strong environmental stress resulted in toxicant effects at approximately 0.0001 µg/L (Fig. 4). Accordingly, small effects of the pesticide (LC_5_) are present at concentrations of approximately 3 to 4 orders of magnitude below those responsible for strong effects (LC_50_). These results are in stark contrast to the results obtained by effect determination with traditional monotonic sigmoidal concentration-response relationships. When SyS is not considered and conventional fitting of the concentration-response is applied, no dependency between environmental stress and low concentration toxicant sensitivity (LC_5_) of individuals could be revealed. We also show that the traditional fitting of concentration-response only identifies a reduction in the LC_5_ compared to the LC_50_ by a factor of 7 (Fig. 4). To facilitate the calculation of low toxicant effects, we provide the EC_x-SyS_ approach as an R-script in the supplementary information and a calculator within the INDICATE program package (http://www.systemecology.eu/indicate/).

## Discussion

When considering a broad range of toxicant concentrations, non-monotonic, tri-phasic concentration-response relationships are often observed. The non-monotonic, bi-phasic relationships^12^, which are currently applied to explain the hormetic increase in survival^4^ at low concentrations, cannot reproduce the observed mortality at sub-hormetic, ultra-low concentrations. Accordingly, the current ecotoxicological cause-response concepts are not capable of predicting small biological effects at low and ultra-low toxicant concentrations. The introduction of “System Stress” (SyS), however, allows the modelling of the tri-phasic concentration-response relationship and a quantification of effects at hormetic and sub-hormetic concentrations. For this we postulate that individuals develop internal SyS over time (Fig. 2). SyS results in the mortality of a small percentage of individuals (Fig. 1A). Pulsed toxicant stress, equivalent to around 0.3 units of general stress reduces SyS to zero (Fig. 2), which in turn increases the survival of individuals (Fig. 1A,B). This concept fundamentally expands current approaches that only associate increased survival under low stress conditions with compensatory responses. We also assume that compensation of toxicant stress increases the survival rate of individuals and their offspring. This was often observed for different groups including aquatic^13^ and terrestrial insects^14^, and crustaceans^15^. However, the increasing mortality under control conditions, without toxicant stress, indicates the presence of a previously unknown but significant presence of stress from a different source. This reasoning inevitably leads to the hypothesis that without the presence of external stress there must be a relevant internal stress at an individual level. An indication of the still unknown processes at an individual level investigated here can be provided by already known processes at population level. Here it is known that an absence of external stress, in combination with a limitation of resources, leads to scramble competition^16^, a possible representation of SyS at population level. Similar to the findings at an individual level presented here, the effect of short-term pesticide stress at population level also shows a tri-phasic concentration-response relationship. For example, low short-term stress may improve survival, generating “population hormesis”. This scenario was observed for the midge *Chironomus riparius* exposed to cadmium^17^, the mayfly *Cloeon dipeterum* exposed to the insecticide esfenvalerate^7^ and the caddisfly *Limnephilus lunatus* exposed to fenvalerate^9^. The common explanation for these observations is that under control conditions, in the absence of a considerable toxicant stress, scramble competition between individuals^16^ increases mortality. In contrast, low toxicant stress reduces population density and accordingly scramble competition the representation of SyS at population level. In addition, all these studies found lower survival compared to control conditions at low, subhormetic concentrations. This reduced survival may be due to a synergistic interaction between very low levels of external stress and SyS, not yet reduced to zero at these low stress levels. The similarities of these concentration-response relationships at population level point to analogous processes at an individual level.

The question remains as to the evolutionary advantage of SyS at an individual level examined here. For populations, high levels of intraspecific competition are relevant drivers of adaptation to changing environmental conditions. For example, stress through intraspecific competition accelerated microevolution towards pesticide resistance in populations of the mosquito *Culex quinquefasciatus* that were exposed to the pesticide chlorpyrifos^18^. Accordingly, we speculate that also for individuals SyS is an expression of the potential to adapt to a changing environment. This would suggest that individuals are evolutionarily “optimized” for a certain amount of short-term stress reflecting the “natural” stress level. The amount of stress at which SyS is reduced to close to zero may therefore be the level of stress typically present in the environment of an individual. Comparably, in the area of human health, a review of the impact of intermittent fasting on health and disease processes revealed that caloric limitation, natural for humans in their evolutionary history, protects against metabolic syndrome and associated disorders including diabetes and cardiovascular disease^19^.

By including “System Stress” and fitting a tri-phasic concentration-effect relationship to measured data with the EC_x-SyS_ approach, it is possible to identify so far unexpected effects of low hormetic and ultra-low sub-hormetic toxicant concentrations. Also the assumption of a threshold concentration is thus questioned. This attribution and quantification of cause and effect is of crucial importance for a more realistic environmental and human risk assessment in the context of environmental stress.

## Methods

### Experiments

We studied the combined effect of the insecticide esfenvalerate and various environmental stressors on *Daphnia magna*. Culture: Individuals were obtained from the clone “Aachen 5”, cultured in Aachener Daphnien medium (ADaM)^20^ Algae were harvested in the exponential growth phase and centrifuged, and the pellets were re-suspended in ADaM to obtain the desired dilutions. Additionally, on weekends, the organisms were fed with 0.6 mg/L yeast. The medium was changed, and neonates were removed daily.

Test organisms were kept individually in 80 mL ADaM medium to avoid intraspecific interactions. The medium (ADaM) was changed every second day, and neonates were removed within 24 h. The microalgae *Desmodesmus subspicatus* was used as a food source. Once a week, 0.25 μg ind.^−1^ yeast was added. Individuals were exposed after 7 days of adaptation to the respective conditions of the pesticide for 24 h and then cultured for an additional 21 days. The mortality of the daphnids was recorded daily, and the dead individuals and neonates were removed daily. For the contaminants, we selected the pyrethroid esfenvalerate (CAS 66230-04-4, purity: 99.8%) at concentrations of 0, 0.03, 0.3, and 3.0 µg/L. We used dimethyl sulfoxide (DMSO) as a solvent for the preparation of the stock solution of esfenvalerate. The DMSO concentration was always kept well below the solvent limit suggested by the Organisation of Economic Cooperation and Development (OECD) guidelines^21^.

### Conditions of treatments for three environmental stressors

Food: During the test, organisms in the treatment with high food amounts were fed 0.5 × 10^9^ cells ind.^−1^ day^−1^ the first week, 1.15 × 10^9^ cells ind.^−1^ day^−1^ the second week, and 1.35 × 10^9^ cells ind.^−1^ day^−1^ the third and fourth weeks. In contrast, organisms in the treatment with low food amounts were fed 0.5 × 10^7^ cells ind.^−1^ day^−1^ the first week, 1.15 × 10^7^ cells ind.^−1^ day^−1^ the second week, and 1.35 × 10^7^ cells ind.^−1^ day^−1^ the third and fourth weeks.

Temperature: Test organisms were kept at 20 °C ± 1 °C (control group) and 30 °C ± 1 °C. During the 24 hours of the contamination pulse, the daphnids from the high temperature treatment were also kept at 20 °C.

UV radiation: Test organisms were exposed to UV-B radiation and visible light in an irradiation chamber (BS-04, version 1.1.0, Opsytec Dr. Gröbel GmbH, Ettlingen, Germany). The chamber contained four UV-B light tubes (broadband TL 20 W/12RS; narrowband 20 W/01RS). Additionally, two UV-A light tubes (LT 20W/05) and two daylight lamps D-65 (Master LT-D 90 Graphica 18 W/965, Philips, The Netherlands) were installed inside the chamber to imitate a realistic radiation spectrum. Two sensors within the chamber measured and displayed the UV-A as well as UV-B dose on the UV-MAT (Version 1.0.4, Opsytec Dr. Gröbel GmbH, Ettlingen, Germany). The UV-B and UV-A intensities at the water surface were 0.2 mW cm^−2^. The test organisms were exposed for 0, 1 and 5 hours every day, resulting in an exposure of 0, 0.72 and 3.6 J cm^−2^ UV, respectively.

The overview of the treatments and the strength of the environmental stressors are given in Table 1.

**Table 1:** Type and strength of environmental stressors applied within the experiments.

Environmental Stressor	Stressor Level	General Environmental Stress	Stress Related Mortality	Average Number of Replicates
UV radiation	0.0 J cm^−^²	0.0	0.0	37
UV radiation	0.72 J cm^−^²	0.034	0.0002	25
UV radiation	3.60 J cm^−^²	0.384	0.284	25
food	100%	0.0	0.0	48
food	1%	0.170	0.033	48
temperature	20 °C	0.0	0.0	48
temperature	30 °C	0.374	0.267	24

### Verification of toxicant concentration

Exposure concentrations of esfenvalerate were analysed by Wessling GmbH, Landsberg OT, Oppin, Germany, using a Thermo Fisher Scientific TSQ™ 8000 Evo Triple Quadrupole GC-MS/MS, Massachusetts, USA. The detection limit of the instrument was 5.7 ng/L using a TG-5HT guard column with a 0.53 mm i.d. and 0.15 μm film thickness (Thermo Fisher Scientific, Hennigsdorf, Germany). The software Trace Finder 3.2 (Thermo Fisher Scientific) was applied for data processing. The measured concentrations of the pesticides in the experimental repetitions are given in Supporting Information (Table S1).

### Calculating with stressors

The identification and calculation of the different stress components is based on the SAM published by Liess *et al*. 2016. The three principal assumptions of the SAM are as follows:

(i) Each individual has a certain capacity to tolerate all types of stress, a general stress capacity, symmetrically distributed over a finite interval [0, 1]. We assume that stress-dependent population sensitivity follows the same distribution. Individuals with a stress capacity below a given stress level S will die, whereas individuals with a stress capacity above a given stress level will survive. Hence, stress-dependent population sensitivity is parameterized by the following beta distribution:
  1 $$p(S)=\frac{1}{B(p,q)}{S}^{p-1}{(1-S)}^{q-1}$$
  where p(S) represents the density probability of individuals to tolerate a general stress S, p and q are the non-negative shape parameters of the distribution and B (p, q) is the beta function, which is a normalization constant to ensure that the total probability integrates to 1. We postulated symmetry of the individual stress capacity (p = q). The parameters were set to p = q = 3.2, which resulted in the best fit between the observed and predicted LC_10_ and LC_50_ shifts in the 23 experimental study pairs in Liess *et al*.^1^. The integral of the density function gives the population size N under non-stress conditions:
  2 $$N={\int }_{0}^{1}p(S)ds=1$$
  The stress-dependent survival is calculated as
  3 $$N(S)=1-{\int }_{0}^{1}p(S)ds$$
  where N(S) = 1 (100% survival) for the general stress S = 0 and N(S) = 0 (0% survival) for the general stress S ≥ 1.
(ii) The SAM assumes that every specific unit of a given stressor can be transferred to a general stress level. This conversion uses stress-related mortality as a linking factor. For instance, if a temperature stress or toxicant stress causes a mortality of 10%, then the general stress level is given by the 10% quantile of the beta distribution in Eq. 1.
(iii) The SAM assumes that the general stress levels of independent stressors are additive, with the sum determining the total general stress exerted on a population. The total general stress S is given as the sum of general stress levels S_i_ of all stressors.

4 $$S=\sum {S}_{i}$$

The resulting survival of the population exposed to the general stress S can be determined by applying Eq. 3. Conversely, its inverse can be used to determine general stress from observed survival. A more detailed description of the approach can be found in the original publication^1^.

### Modelling the tri-phasic concentration-response relationship

(i) The toxicant-related mortality without environmental stress is estimated. For this, we assume that the concentration-response relationship follows a Weibull function. The shape of this relationship is determined by the observed survival at high concentrations from full mortality until hormesis (see Fig. 1A), at a range of concentrations where no SyS is present and, additionally, at the control, where no toxicant stress is present. Accordingly, the modelling of the toxicant-related concentration-response relationship is generally determined with four measured values. This process allows an accurate determination of monotonic toxicant-related survival (Fig. 1A) by fitting the Weibull function given in Eq. 5, as implemented in R by the drc package^22^. The upper limit d was fixed at 1 (100% survival), and the lower limit c was fixed at 0 (0% survival). Least squares optimization resulted in b = 1.256 and e = 2.877 for the example data (see the dashed red line in Fig. 1A). This modelled survival was then converted into the toxicant-related stress via Eq. 3.
  5 $$f(x)=c+(d-c)\exp (-\exp (b(\log (x)-\,\log (e))))$$
  where b represents the relative slope around the inflection point; c and d are the lower and upper limits, respectively; and e is the inflection point^23^. The variable x represents the toxicant concentration when modelling survival under toxicant stress alone. When the function is used to model the SyS, x represents the toxicant stress.
(ii) the Weibull function described in (i) tends to overshoot in the hormetic concentration range. To archive a more reasonable and realistic curve shape three smoothing data points are approximated by linear interpolation on a logarithmic scale between the sub-hormetic and the hormetic concentration.
(iii) the System Stress (SyS) without environmental stress is estimated by assuming that in the control all mortality is induced by SyS. At the hormesis concentration, and above, we assume no SyS-related mortality. SyS is reduced with increasing S_Tox_ described by a Weibull function. Applying Eq. 6 we obtain S_SyS_.
  6 $${S}_{SyS}={S}_{Obs}-{S}_{Tox}$$
  S_Obs_ is the observed mortality; S_Tox_ is calculated according to (i). Then S_SyS_ and S_Tox_ are fitted with a least squares optimization according to Eq. 5 with the lower limit fixed at 0. From this, we obtain b = 3.476, d = 0.299 and e = 0.152. This process enables us to estimate S_SyS_ for the whole range of concentrations (see the dashed blue line in Fig. 1B). SyS is also used to define the hormetic range – the concentration where Sys is reduced to 50%, up to the concentration where SyS is 1% and thus negligible (Fig. 1A,C).
(iv) The combined stress (S_Sum_) in the absence of environmental stress is determined by adding toxicant stress (S_Tox_) and system stress (S_SyS_) according to SAM (Eq. 7).
  7 $${S}_{Sum}={S}_{SyS}+{S}_{Tox}$$
  The resulting stress (S_Sum_) is converted into the modelled survival (Fig. 1A).
(v) The intensity of additional environmental stress can be determined by comparing the survival in an experiment without and with environmental stress at the hormesis concentration, where SyS is close to 0 – the EC_99_ of the SyS (Eq. 8). At this concentration, the observed difference in survival can be attributed to environmental stress (S_Env_). This concentration is best suited for the determination of S_Env_ as the S_Tox_ is minimal without the presence of SyS.
  8 $${S}_{Env}={S}_{Obs,Hormesis}-{S}_{Tox,Hormesis}$$
  where S_Obs,Hormesis_ and S_Tox,Hormesis_ are the corresponding stress levels at the hormesis concentration and the EC_99_ of the SyS, respectively.
(vi) System stress in the presence of an environmental stressor.
  The sum of the S_Env_ and the S_Tox_ is the external stress S_EXT_:
  9 $${S}_{Ext}={S}_{Env}+{S}_{Tox}$$
  The difference between the total observed stress and this external stress is the system stress S_SyS_:
  10 $${S}_{SyS}={S}_{Obs}-{S}_{Ext}$$
  The relationship between this System Stress and S_Tox_ is then fitted with the Weibull function given in Eq. 5. The lower limit c was again fixed at 0, and the least squares optimization resulted in b = 2.281, d = 0.256, and e = 0.133 for the example data (see the red line in Fig. 2). This system stress is different from the one calculated in the experiment without the environmental stressor. However, environmental stress does not reduce system stress, the reason to use S_Tox_ instead of S_Ext_ for this fit.
(vii) The sum of system stress and external stress is the modelled total stress S:

11 $$S={S}_{SyS}+{S}_{Ext}$$

The resulting total stress can be converted to give the modelled survival.

There may be experiments that do not show an obvious hormesis, even where low concentrations were tested. In our data set, this can be seen for the experiment that includes low food amounts as an additional environmental stressor (Fig. SI 1C). The reason for this result may be that the environmental stressor acts highly synergistically with the toxicant so that the absence of SyS at the hormesis point does not result in increased survival. Nevertheless, the shape of the figure shows that mortality increases at ultra-low concentrations; at low concentrations, survival does not increase as expected with a logistic response relationship. However, determining the hormesis point is more difficult in such cases. For these cases, we suggest identifying the “step” from which survival rates rapidly decrease in the concentration-response relationship and allocating hormesis to this concentration. A prerequisite for the application of the approach is that the observed survival rate in the control will not exceed 100%, i.e., the individuals should not reproduce. Model development and data analysis were performed with R 3.5^24^ and RStudio 1.2^25^, respectively.

## Supplementary information

Supplementary Information:

Supplementary Dataset 1

## Data Availability

All data and analysis code are publicly available.
