# Supplementary material for: Predicting low-concentration effects of pesticides
Source: Sci Rep. 2019 Oct 24;9:15248. doi: 10.1038/s41598-019-51645-4 (PMC6813311; doi:10.1038/s41598-019-51645-4)
Supplement: Supplementary file 1 — Supplementary Information: [file 41598_2019_51645_MOESM1_ESM.pdf]

Supplementary Information:

Predicting low-concentration effects of pesticides

Matthias Liess[1,2] *, Sebastian Henz[1,2], Saskia Knillmann[1]

[1] UFZ - Helmholtz Centre for Environmental Research, Dept. of System-Ecotoxicology, Permoserstrasse 15, D-04318 Leipzig, Germany
[2] RWTH Aachen University, Institute for Environmental Research (Biology V), Worringerweg 1, 52074 Aachen, Germany

[*]Corresponding author, matthias.liess@ufz.de

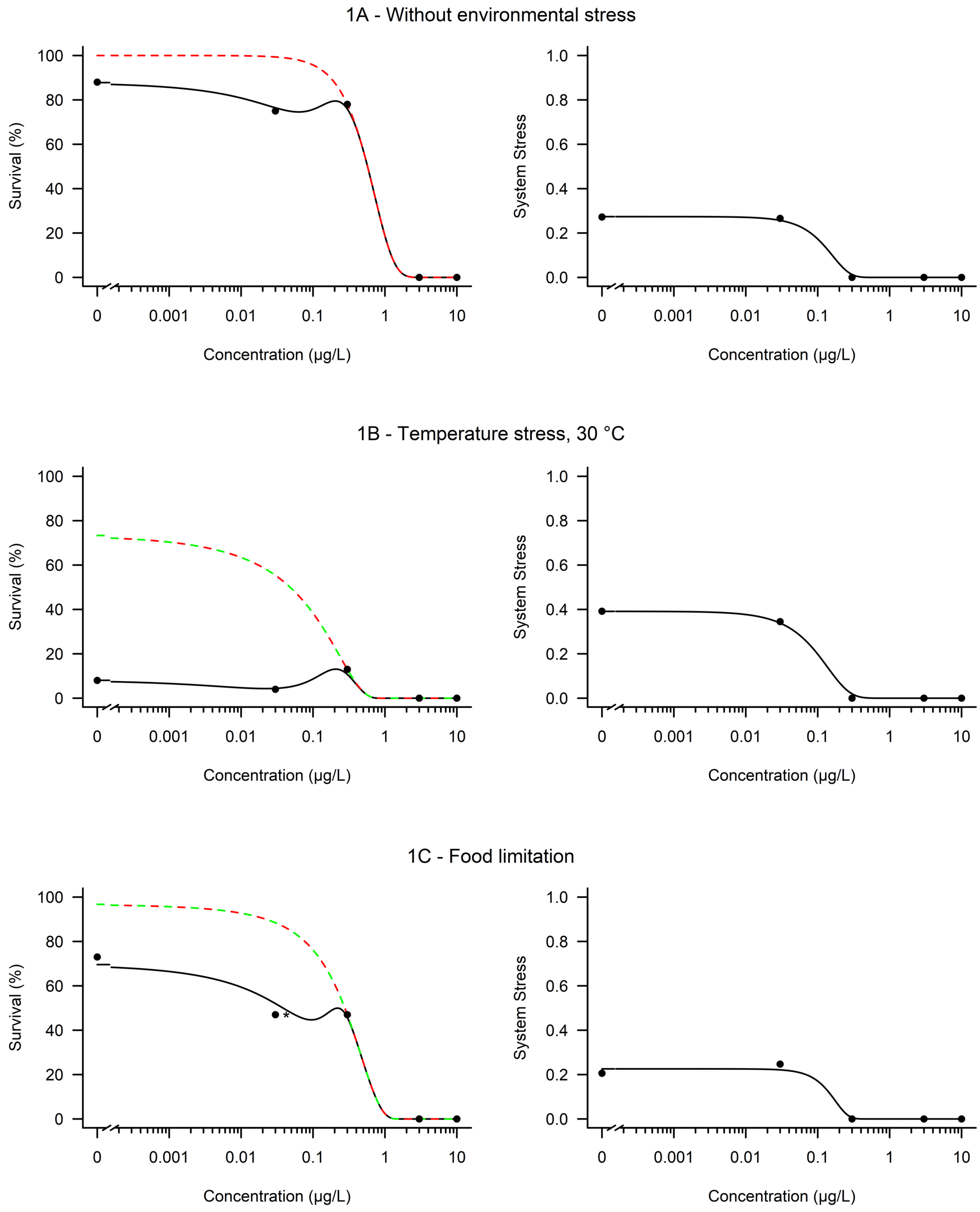

Figure S1 | Experiments with no additional stressor, UV-radiation, temperature, and food limitation. – continued –

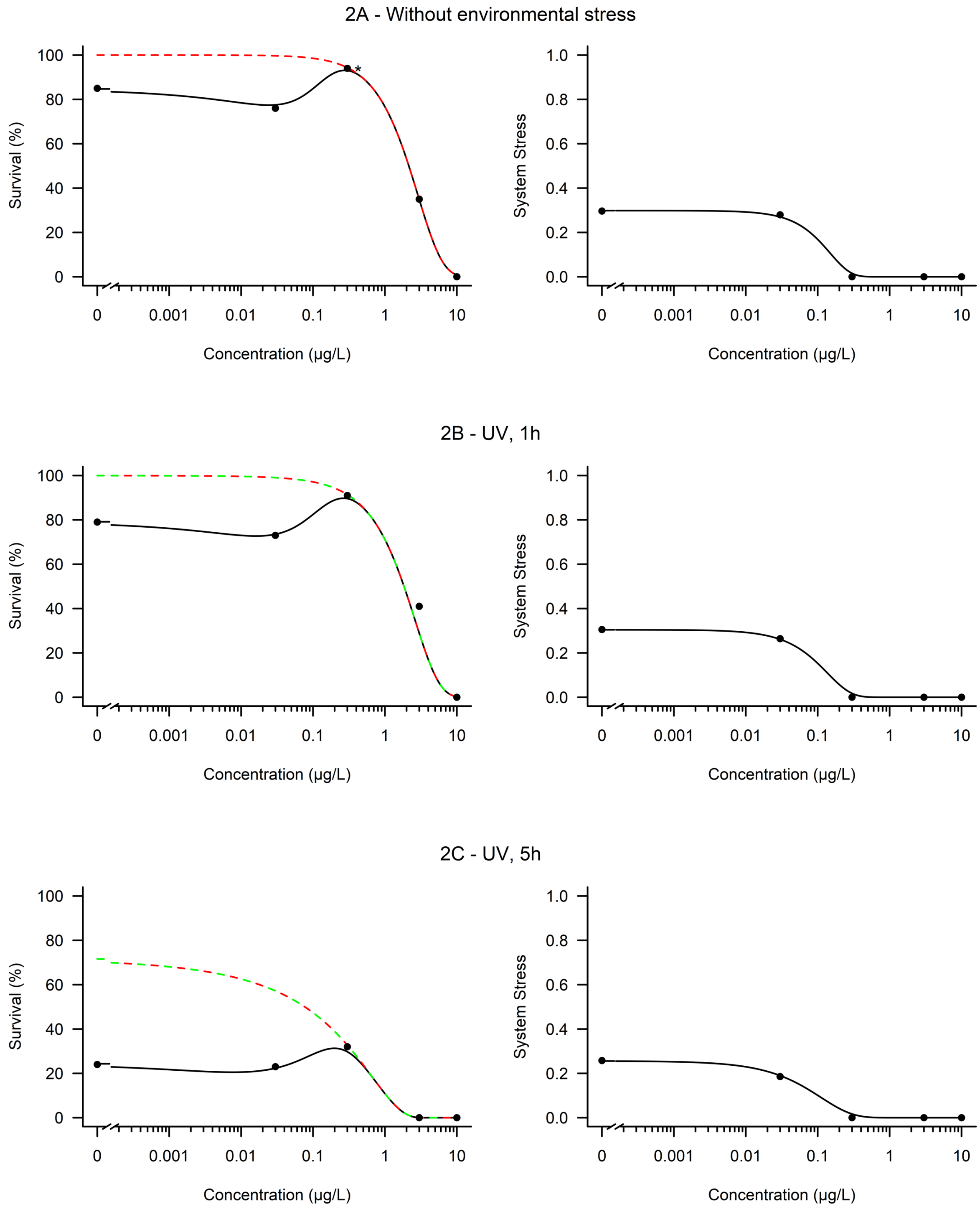

Figure S1 | Experiments with no additional stressor, UV-radiation, temperature, and food limitation. A) Observed mortality (circles), modelled concentration-response relationship (line), toxicant-related survival (dashed line, red), toxicant- and environmental stress-related survival (dashed line, green and red, respectively). **B)** System stress (SyS). Modelled at observed mortality (circles) and modelled stress-response relationship (line).

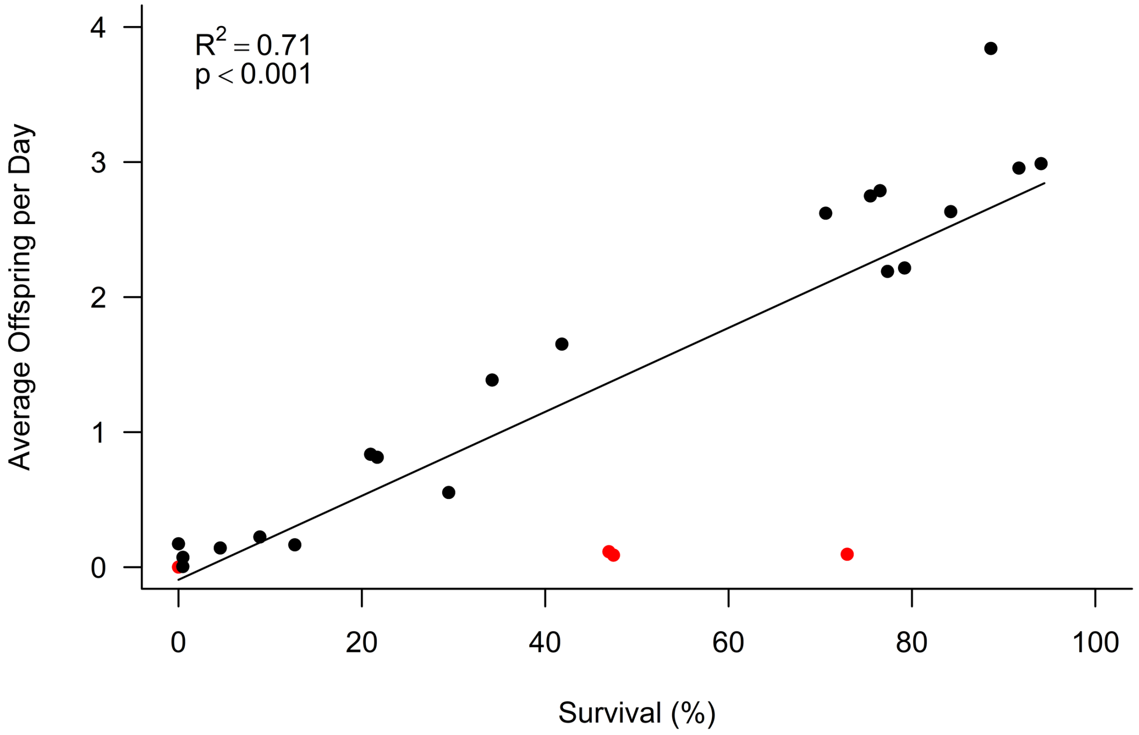

Figure S2 | Relation survival and offspring. Offspring number per surviving individual per day in relation to percentage survival of individuals in each treatment. Red dots are from the low-food experiment. Overlapping dots were manually separated to show all data points in the plot.

Table S1 | Measured concentrations of the insecticide esfenvalerate.

experiment	Nominal conc. (µg L^{-1})	Measured conc. (µg L^{-1})
UV radiation	Control	Not detected
	0.03	0.027
	0.03	0.025
	0.3	0.31
	0.3	0.12
	3	3.8
	3	3.6
Temperature, Low Food	Control	Not detected
	0.03	0.019
	0.03	0.036
	0.3	0.21
	0.3	0.26
	3	2
	3	2.7

Table S2 | Comparison of the goodness of fit of the traditional log-logistic concentration response model and the $EC_{x\text{-}SyS}$ approach. For each experiment the goodness of fit was calculated as the sum of squared errors (SSE), using (i) predictions from a traditional log-logistic model and (ii) of the $EC_{x\text{-}SyS}$ approach. The SSE´s of the $EC_{x\text{-}SyS}$ approach are consistently smaller than those of the traditional model, indicating a better fit. This difference is significant with $p < 0.01$ (one-tailed paired t-test).

experiment	SSE, traditional model	SSE, $EC_{X\text{-}SyS}$
1A – Without environmental stress	240.61	6.50
1B – Temperature stress, 30 °C	41.09	5.42
1C – Food limitation	411.78	38.28
2A – Without environmental stress	315.64	3.58
2B – UV, 1h	280.05	146.51
2C – UV, 5h	65.91	7.47
